# ﻿Morphological and phylogenetic evidence reveals three new arthropod-associated species of Hypocreales (Clavicipitaceae, Bionectriaceae, and Myrotheciomycetaceae) from karst habitats in Guizhou, China

**DOI:** 10.3897/mycokeys.123.164334

**Published:** 2025-10-17

**Authors:** Wan-Hao Chen, Hui-Lin Shu, Dan Li, Jian-Dong Liang, Xiu-Xiu Ren, Nalin N. Wijayawardene, Yan-Feng Han, Jie-Hong Zhao

**Affiliations:** 1 Center for Mycomedicine Research, Basic Medical School, Guizhou University of Traditional Chinese Medicine, Guiyang 550025, Guizhou Province, China; 2 Institute of Fungus Resources, Department of Ecology, College of Life Sciences, Guizhou University, Guiyang 550025, Guizhou Province, China; 3 Key Laboratory of Microbio and Infectious Disease Prevention & Control in Guizhou Province, Guiyang 550025, Guizhou Province, China; 4 College of Pharmacy, Guizhou University of Traditional Chinese Medicine, Guiyang 550025, Guizhou Province, China; 5 Center for Yunnan Plateau Biological Resources Protection and Utilization, College of Biological Resource and Food Engineering, Qujing Normal University, Qujing, Yunnan 655011, China; 6 Tropical Microbiology Research Foundation, 96/N/10, Meemanagoda Road, Pannipitiya 10230, Sri Lanka

**Keywords:** Insect, karst, morphology, phylogenetic analysis, spider

## Abstract

The karst regions of southwest China are rich in biodiversity and have critically threatened ecosystems, harboring unique species that could be new to science. During the investigations of arthropods associated-fungi, several fungal strains were collected. Among these, three new species, *Conoideocrella tiankengensis***sp. nov.** (Clavicipitaceae), *Ovicillium zunyiense***sp. nov.** (Bionectriaceae) and *Trichothecium sinense***sp. nov.** (Myrotheciomycetaceae), isolated from a dead scale insect, larva and spider, respectively, were introduced as novel taxa, based on the morphological characteristics and DNA-based phylogenetic analyses. This is the first time that a species from Myrotheciomycetaceae is reported from the karst habitats. In addition, the genus *Myrotheciomyces* is treated as a synonym of *Trichothecium* based on the phylogenetic analysis, and the type species of the former is transferred to the latter genus.

## ﻿Introduction

Karst regions, particularly those in southwest China, harbor vast tracts of well-preserved primary forests with exceptionally high biodiversity (Wijayawardene et al. 2021; Sun et al. 2023, 2024). The intricate ecosystems and unique geological conditions of these areas serve as important microhabitats for numerous ancient species (Özkan et al. 2010; Su et al. 2017). Moreover, the region is now recognized as one of the most critically threatened biodiversity hotspots globally due to the unique and highly fragile nature of its ecosystem and anthropogenic activities (Long et al. 2024). Hence, urgent conservation measures are needed to protect these vulnerable ecosystems (Wu and Zhang 2020; Duan et al. 2021).

Guizhou Province, a quintessential karst region in China, is characterized by its crisscrossing mountain ranges and dramatic elevation variations. These topographical contrasts have fostered diverse microenvironments, shaping intricate ecosystems that support a wide array of species across distinct habitats (Liu et al. 2018). As a key hub of biodiversity, Guizhou Province hosts exceptional ecological richness (Xu et al. 2017).

Wijayawardene et al. (2021) regarded the Guizhou and Yunnan Provinces as mycological hotspots. Annually, a large number of novel fungal species are introduced from different substrates, including arthropods, and these taxa could be parasitic or saprobes of insects and spiders (e.g. Chen et al. 2024a, b, d). Recent studies reported new arthropod-associated fungi that are accommodated in nine families (For a list of the species, see Suppl. material 1), *viz.*, Bionectriaceae (Chen et al. 2024a; Wang et al. 2025a), Calcarisporiaceae (Chen et al. 2024b), Clavicipitaceae (Chen et al. 2022a), Cordycipitaceae (Chen et al. 2023; Bu et al. 2025), Hypocreaceae (Tarafder et al. 2024), Nectriaceae (Wang et al. 2024), Ophiocordycipitaceae (Chen et al. 2024c; Peng et al. 2024; Xu et al. 2025), Polycephalomycetaceae (Chen et al. 2024c), Tilachlidiaceae (Liang et al. 2016) from Guizhou Province.

During a survey of arthropod-associated species in Hypocreales from southwestern China, several specimens were collected, and fungal strains were isolated and purified. Isolated strains were identified based on the multigene phylogeny and morphological characteristics, and three new species introduced, i.e. *Conoideocrella tiankengensis* sp. nov., *Ovicillium zunyiense* sp. nov. and *Trichothecium sinense*sp. nov., which belong to the families Clavicipitaceae, Bionectriaceae and Myrotheciomycetaceae, respectively. This is the first report of a taxon from the family Myrotheciomycetaceae reported in the Guizhou karst habitats. Moreover, the type species of *Myrotheciomyces*, *M. corymbiae* resided in *Trichotheciums. str.* Thus, *Myrotheciomyces* is regarded as a synonym of *Trichothecium*.

## ﻿Materials and methods

### ﻿Specimen collection and isolation

The specimens were collected from Monkey-Ear Tiankeng (27°5'12.138"N, 107°40'48.42"E), Kaiyang County, Guiyang City, Mayao River Valley (26°21'24.71"N, 107°22'48.22"E), Duyun City, Qiannan Buyi and Miao Autonomous Prefecture and Dabanshui National Forest Park (27°46'35.904"N, 106°48'30.89"E), Honghuagang District, Zunyi City, Guizhou Province, on 6^th^ April 2024, 1^st^ May 2022 and 2^nd^ September 2023, respectively. The samples were placed in sterile bags, kept separately on ice, and transported to the laboratory. Specimens were preserved in the refrigerator at 4 °C until further processing.

The surface of each arthropod body was rinsed with sterile water, followed by sterilization with 75% ethanol for 3–5 s and rinsing again three times with sterilized water. After drying on sterilized filter paper, a piece of the mycelium or sclerotium was cut from the specimen and placed on plates of potato dextrose agar (PDA) (Potato powder 6%, Agar 20%, Glucose 20%, Beijing Solarbio Technology Co., Ltd., China) or PDA modified by the addition of 1% w/v peptone (Beijing Solarbio Technology Co., Ltd., China) containing 0.1 g/l streptomycin (Beijing Solarbio Technology Co., Ltd., China) and 0.05 g/l tetracycline (Beijing Solarbio Technology Co., Ltd., China) (Chen et al. 2019). After fungal colonies emerged from the plated samples, a piece of mycelium from the colony edge was transferred onto new agar plates and cultured at 25 °C for 14 days under 12 h light/12 h dark conditions (Zou et al. 2010).

### ﻿Morphological study

Colony characteristics were determined on PDA cultures incubated at 25 °C for 14 days, and growth rate, presence of octahedral crystals and colony colors (surface and reverse) were observed. To investigate microscopic characteristics, a little of the mycelia was picked up from the colony and mounted in lactophenol cotton blue or 20% lactic acid solution and the asexual morphological characteristics (e.g., conidiophores, phialides or conidiogenous cells, and conidia) were observed and measured using a Leica DM4 B microscope.

### Maintenance and deposition of type materials and registration of novel taxa

The holotypes and ex-type cultures were deposited at the Institute of Fungus Resources, Guizhou University (formerly Herbarium of Guizhou Agricultural College; code, GZAC), Guiyang City, Guizhou, China. MycoBank numbers were obtained for novel taxa as outlined in MycoBank (http://www.MycoBank.org) (Crous et al. 2004).

### ﻿DNA extraction, polymerase chain reaction amplification (PCR) and nucleotide sequencing

DNA extraction was carried out using a fungal genomic DNA extraction kit (DP2033, BioTeke Corporation) according to Liang et al. (2011). The extracted DNA was stored at −20 °C. Polymerase chain reaction (PCR) was used to amplify genetic markers using the following primer pairs: ITS5/ITS4 for the internal transcribed spacer (ITS) region (White et al. 1990), LR0R/LR5 for 28S large subunit ribosomal RNA gene (LSU) (Vilgalys and Hester 1990), fRPB2-5F/fRPB2-7cR for RNA polymerase II second largest subunit (*RPB2*) (Liu et al. 1999) and 983F/2218R for translation elongation factor 1 alpha (*tef*-1α) (Castlebury et al. 2004). The thermal cycle conditions of PCR amplification for these phylogenetic markers were set up following the procedure described by Chen et al. (2021) (Table 1). The PCR products were purified and sequenced at Sangon Biotech (Shanghai) Co. All newly generated sequences were deposited in GenBank and accession numbers were obtained (Table 2).

**Table 1.** List of primer information used in this study.

Locus	Primer	Length	Direction	Sequence 5’-3’	Optimised PCR protocols	References
ITS	ITS5	22	forward	GGAAGTAAAAGTCGTAACAAGG	(95 °C: 30 s, 51 °C: 50 s, 72 °C:45 s) × 33 cycles	White et al. 1990
	ITS4	20	reverse	TCCTCCGCTTATTGATATGC		
LSU	LROR	17	forward	ACCCGCTGAACTTAAGC	(94 °C: 30 s, 51 °C: 1 min,72 °C: 2 min) × 33 cycles	Vilgalys and Hester 1990
	LR5	17	reverse	TCCTGAGGGAAACTTCG		
*RPB2*	RPB2-5F3	20	forward	GACGACCGTGATCACTTTGG	(94 °C: 30 s, 54 °C: 40 s, 72 °C:1 min 20 s) × 33 cycles	Liu et al. 1999
	RPB2-7Cr2	20	reverse	CCCATGGCCTGTTTGCCCAT		
*tef*-1α	983F	23	forward	GCYCCYGGHCAYCGTGAYTTYAT	(94 °C: 30 s, 58 °C: 1 min 20 s,72 °C: 1 min) × 33 cycles	Castlebury et al. 2004
	2218R	23	reverse	ATGACACCRACRGCRACRGTYTG		

**Table 2.** List of strains and GenBank accession numbers of sequences used in this study.

Species	Strain No.	GenBank Accession No.				Reference
		ITS	LSU	*RPB2*	*tef*-1α	
*Acremonium alternatum*	CBS 407.66^T^	OQ429442	OQ055353	OQ560696	OQ470739	Hou et al. 2023
*A. egyptiacum*	CBS 114785^T^	OQ429456	OQ055362	OQ453845	OQ470749	Hou et al. 2023
*Akanthomyces aculeatus*	HUA 772	KC519371	-	-	KC519366	Sanjuan et al. 2014
*Albacillium hingganense*	SGSF339^T^	OR740562	OR740566	OR769081	MN065771	Ding et al. 2024
*Bionectria ochroleuca*	AFTOL-ID 187	-	DQ862027	DQ862013	DQ862029	Zhang et al. 2006
*B. vesiculosa*	HMAS 183151^T^	HM050304	HM050302	-	-	Luo and Zhuang 2010
*Bulbithecium arxii*	CBS 737.84 ^T^	OQ429505	OQ055416	OQ451834	OQ470794	Hou et al. 2023
*B. borodinense*	CBS 101148 ^T^	OQ429506	OQ055417	-	OQ470795	Hou et al. 2023
*B. pinkertoniae*	CBS 157.70 ^T^	OQ429509	OQ055420	OQ453898	OQ470799	Hou et al. 2023
*B. spinosum*	CBS 136.33 ^T^	OQ429512	OQ055423	OQ453899	OQ470802	Hou et al. 2023
*Calcarisporium arbuscula*	CBS 221.73^T^	AY271809	-	-	-	Sun et al. 2016
*C. arbuscula*	CBS 900.68	KT945003	KX442598	KX442597	KX442596	Sun et al. 2017
*C. cordycipiticola*	CGMCC 3.17905^T^	KT944999	KX442599	KX442594	KX442593	Sun et al. 2016
*C. cordycipiticola*	CGMCC 3.17904	KT945001	KX442604	KX442607	KX442605	Sun et al. 2016
*C. xylariicola*	HMAS 276836^T^	KX442603	KX442601	KX442606	KX442595	Sun et al. 2017
*Calonectria ilicicola*	CBS 190.50	GQ280605	GQ280727	KM232307	AY725726	Lombard et al. 2010
*Cephalosporium curtipes*	CBS 154.61	AJ292404	AF339548	EF468947	EF468802	Sung et al. 2007
*Claviceps fusiformis*	ATCC 26019	JN049817	U17402	-	DQ522320	Spatafora et al. 2007
*Clonostachys phyllophila*	CBS 921.97^T^	AF210664	OQ055445	OQ453921	OQ470826	Hou et al. 2023
*C. rosea*	GJS90-227	-	AY489716	-	AY489611	Castlebury et al. 2004
*C. spinulosispora*	CBS 133762^T^	MH634702	KY006568	-	-	Zhao et al. 2025
*Cocoonihabitus sinensis*	HMAS254523^T^	KY924870	KY924869	-	-	Zhuang and Zeng 2017
*C. sinensis*	HMAS254524	MF687395	MF687396	-	-	Zhuang and Zeng 2017
*Conoideocrella fenshuilingensis*	YHH CFFSL2310002^T^	-	PP178583	-	PP776168	Wang et al. 2024
*C. fenshuilingensis*	YHH CFFSL2310003	-	PP178584	-	PP776169	Wang et al. 2024
*C. gongyashanensis*	CGMCC 3.28305^T^	-	PQ278801	PQ334678	PQ301442	Lin et al. 2025
*C. gongyashanensis*	CGMCC 3.28306	-	PQ278802	PQ334679	PQ301443	Lin et al. 2025
*C. krungchingensis*	BCC 36100^T^	-	KJ435080	-	KJ435097	Mongkolsamrit et al. 2016
*C. krungchingensis*	BCC 36101	-	KJ435081	-	KJ435098	Mongkolsamrit et al. 2016
*C. luteorostrata*	NHJ 11343	-	EF468850	-	EF468801	Sung et al. 2007
*C. luteorostrata*	NHJ 12516	-	EF468849	EF468946	EF468800	Sung et al. 2007
*C. tenuis*	NHJ 6791	-	EU369046	EU369089	EU369028	Johnson et al. 2009
*C. tenuis*	NHJ 6293	-	EU369044	EU369087	EU369029	Johnson et al. 2009
*C. tenuis*	NHJ 345.01	-	EU369045	EU369088	EU369030	Johnson et al. 2009
*C. tiankengensis*	KY04071^T^	PV688356	PV688364	PV705684	PV705692	This study
*C. tiankengensis*	KY04072	PV688357	PV688365	PV705685	PV705693	This study
*Cordyceps brongniartii*	BCC16585	JN049867	JF415967	JF415991	JF416009	Sung et al. 2007
*C. militaris*	OSC93623^T^	JN049825	AY184966	-	DQ522332	Sung et al. 2007
*Dactylonectria alcacerensis*	CBS 129087	JF735333	KM231629	-	JF735819	Cabral et al. 2012
*Elaphocordyceps ophioglossoides*	NBRC 106332^T^	JN943322	JN941409	-	-	Schoch et al. 2012
*E. paradoxa*	NBRC 106958	JN943324	JN941411	-	-	Schoch et al. 2012
*Emericellopsis brunneiguttula*	CBS 111360^T^	OQ429545	OQ055457	OQ453932	OQ470838	Hou et al. 2023
*E. microspora*	CBS 380.62^T^	OQ429567	OQ055481	OQ453967	OQ470875	Hou et al. 2023
*E. salmosynnemata*	CBS 182.56^T^	OQ429579	OQ055492	OQ453977	OQ470887	Hou et al. 2023
*E. terricola*	CBS 120.40^T^	OQ429582	OQ055495	OQ453980	OQ470890	Hou et al. 2023
*Epichloe typhina*	ATCC 56429	JN049832	U17396	DQ522440	AF543777	Spatafora et al. 2007
*Flammocladiella aceris*	CPC 24422^T^	KR611883	KR611901	-	-	Crous et al. 2015
*Fusarium circinatum*	CBS 405.97	U61677	-	JX171623	KM231943	Lombard et al. 2015
*F. sublunatum*	CBS 189.34	HQ897830	KM231680	-	-	Lombard et al. 2015
*Gelasinospora tetrasperma*	AFTOL-ID 1287^T^	-	DQ470980	DQ470932	DQ471103	Spatafora et al. 2006
*Haptocillium sinense*	CBS 567.95	AJ292417	AF339545	-	-	Sun et al. 2017
*Hydropisphaera erubescens*	ATCC 36093^T^	-	AF193230	AY545731	DQ518174	Sun et al. 2017
*H. lutea*	ATCC 208838	-	AF543791	DQ522446	AF543781	Sun et al. 2017
*H. peziza*	GJS92-101^T^	-	AY489730	-	AY489625	Sun et al. 2017
*H. rufa*	DAOM JBT1003	JN942883	JN938865	-	-	Sun et al. 2017
*Hypocrea americana*	AFTOL-ID 52	DQ491488	AY544649	-	DQ471043	Sun et al. 2017
*Hypocrella discoidea*	BCC 8237	JN049840	DQ384937	DQ452461	DQ384977	Sun et al. 2017
*Hypomyces polyporinus*	ATCC 76479	-	AF543793	-	AF543784	Sun et al. 2017
*Lecanicillium attenuatum*	CBS 402.78	AJ292434	AF339565	EF468935	EF468782	Sun et al. 2017
*L. lecanii*	CBS 101247	JN049836	KM283794	KM283859	DQ522359	Sun et al. 2017
*L. psalliotae*	CBS 367.86	-	KM283800	-	KM283823	Park et al. 2015
*Metapochonia gonioides*	CBS 891.72^T^	AJ292409	AF339550	DQ522458	DQ522354	Sun et al. 2017
*Metarhiziopsis microspora*	CEHS133a	EF464589	EF464571	-	-	Li et al. 2008
*M. microspora*	INEHS133a	EF464583	EF464572	-	-	Li et al. 2008
*Metarhizium anisopliae*	CBS 130.71^T^	MT078884	MT078853	MT078918	MT078845	Sung et al. 2017
*M. flavoviride*	CBS 125.65	MT078885	MT078854	MT078919	MT078846	Sung et al. 2017
*M. flavoviride*	CBS 218.56^T^	MH857590	MH869139	-	KJ398787	Sung et al. 2017
*Myrotheciomyces corymbiae*	CPC 33206^T^=CBS 144420	NR_160351	NG_064542	-	-	Crous et al. 2018
*M. corymbiae*	CBS 144420	-	OR052125	-	OQ471031	Hou et al. 2023
*Myrothecium inundatum*	IMI158855^T^	-	AY489731	-	AY489626	Castlebury et al. 2004
*M. roridum*	ATCC 16297	-	AY489708	-	AY489603	Castlebury et al. 2004
*M. verrucaria*	ATCC 9095	-	AY489713	-	AY489608	Castlebury et al. 2004
*Nectria cinnabarina*	CBS 125165	HM484548	HM484562	KM232402	HM484527	Sun et al. 2017
*N. nigrescens*	CBS 125148^T^	HM484707	HM484720	KM232403	HM484672	Sun et al. 2017
*Nectriopsis violacea*	CBS 424.64^T^	-	AY489719	-	-	Castlebury et al. 2004
*Neoaraneomyces araneicola*	DY101711^T^	MW730520	MW730609	MW753026	MW753033	Chen et al. 2022
*N. araneicola*	DY101712	MW730522	MW730610	MW753027	MW753034	Chen et al. 2022
*Neobarya parasitica*	Marson s/n^T^	KP899626	KP899626	-	-	Lawrey et al. 2015
*Neonectria candida*	CBS 151.29	JF735313	AY677333	-	JF735791	Sun et al. 2017
*N. faginata*	CBS 217.67	HQ840385	HQ840382	DQ789797	JF268746	Sun et al. 2017
*N. neomacrospora*	CBS 118984	HQ840388	HQ840379	DQ789810	JF268754	Sun et al. 2017
*N. ramulariae*	CBS 182.36^T^	HM054157	HM042435	DQ789793	HM054092	Sun et al. 2017
*Neurospora crassa*	ICMP 6360	AY681193	AY681158	-	-	Cai et al. 2006
*Niesslia exilis*	CBS 560.74	-	AY489720	-	AY489614	Castlebury et al. 2004
*Ophiocordyceps heteropoda*	EFCC 10125	JN049852	EF468812	EF468914	EF468752	Sung et al. 2007
*O. sinensis*	EFCC 7287	JN049854	EF468827	EF468924	EF468767	Sung et al. 2007
*O. stylophor*	OSC 111000	JN049828	DQ518766	DQ522433	DQ522337	Sung et al. 2007
*Orbiocrella petchii*	NHJ 6240	-	EU369038	EU369082	EU369022	Johnson et al. 2009
*O. petchii*	NHJ 6209	-	EU369039	EU369081	EU369023	Johnson et al. 2009
*O. petchii*	NHJ 5318	-	EU369040	EU369080	EU369021	Johnson et al. 2009
*Ovicillium asperulatum*	CBS 130362^T^	OQ429756	OQ055655	OQ454167	OQ471082	Hou et al. 2023
*O. asperulatum*	CBS 426.95	KU382192	KU382233	OQ454166	OQ471081	Hou et al. 2023
*O. attenuatum*	CBS 399.86^T^	OQ429757	OQ055656	OQ454168	OQ471083	Hou et al. 2023
*O. attenuatum*	CBS 112092	PV272703	PV272923	-	PV273483	Hou et al. 2023
*O. oosporum*	CBS 110151^T^	OQ429758	OQ055657	OQ454169	OQ471084	Hou et al. 2023
*O. oosporum*	CBS 403.89	PV272717	PV272937	PV273301	PV273497	Hou et al. 2023
*O. pseudoattenuatum*	GMBC 3007^T^	PQ726817	PQ726842	PQ779073	PQ758607	Wang et al. 2025
*O. pseudoattenuatum*	GMBC 3008	PQ726818	PQ726843	PQ779074	PQ758608	Wang et al. 2025
*O. sinense*	SD09701^T^	PP836762	PP836764	-	PP852887	Chen et al. 2024
*O. sinense*	SD09702	PP836763	PP836765	-	PP852888	Chen et al. 2024
*O. subglobosum*	CBS 101963^T^	OQ429759	OQ055658	OQ454170	OQ471085	Hou et al. 2023
*O. subglobosum*	CBS 578.89	PV272705	PV272925	PV273289	PV273485	Zhao et al. 2025
*O. theobromae*	CBS 110153^T^	PV272706	PV272926	PV273290	PV273486	Zhao et al. 2025
*O. theobromae*	CBS 119658	PV272707	PV272927	PV273291	PV273487	Zhao et al. 2025
*O. variecolor*	CBS 130360	OQ429760	OQ055659	OQ454171	OQ471086	Hou et al. 2023
*O. variecolor*	CBS 535.81	PV272708	PV272928	PV273292	PV273488	Zhao et al. 2025
*O. zunyiense*	ZY09271^T^	PV688358	PV688366	PV705686	PV705694	This study
*O. zunyiense*	ZY09272	PV688359	PV688367	PV705687	PV705695	This study
*Paraneoaraneomyces sinensis*	ZY 22.006	OQ709254	OQ709260	OQ719621	OQ719626	Hou et al. 2023
*P. sinensis*	ZY 22.007	OQ709255	OQ709261	OQ719622	OQ719627	Hou et al. 2023
*P. sinensis*	ZY 22.008^T^	OQ709256	OQ709262	OQ719623	OQ719628	Hou et al. 2023
*Parasarocladium breve*	CBS 150.62^T^	OQ429781	OQ055677	OQ454192	OQ471107	Hou et al. 2023
*P. chondroidum*	CBS 652.93^T^	OQ429785	OQ055681	OQ454196	OQ471111	Hou et al. 2023
*P. debruynii*	CBS 144942^T^	MK069420	MK069416	OQ454197	-	Hou et al. 2023
*P. funiculosum*	CBS 141.62 ^T^	OQ429786	OQ055682	OQ454198	OQ471307	Hou et al. 2023
*P. gamsii*	CBS 726.71 ^T^	OQ429787	OQ055683	OQ454199	OQ471112	Hou et al. 2023
*P. radiatum*	CBS 142.62 ^T^	OQ429788	OQ055684	OQ454200	OQ471308	Hou et al. 2023
*Peethambara spirostriata*	CBS110115	-	AY489724	EF692516	AY489619	Sun et al. 2017
*Pleurocordyceps aurantiaca*	MFLUCC 17-2113	MG136916	MG136910	-	MG136875	Xiao et al. 2018
*P. marginaliradians*	MFLU 17-1582 ^T^	MG136920	MG136914	-	MG136878	Xiao et al. 2018
*Polycephalomyces albiramus*	GACP 21-XS08T	OQ172092	OQ172037	OQ459807	OQ459735	Xiao et al. 2023
*P. formosus*	NBRC 109993T	MN586833	MN586842	MN598064	MN598057	Xiao et al. 2023
*Proxiovicillium blochii*	CBS 427.93 ^T^	OQ429816	OQ430079	OQ454213	OQ471144	Hou et al. 2023
*P. blochii*	CBS 324.33	OQ429815	OQ430078	OQ454212	OQ471143	Hou et al. 2023
*P. lepidopterorum*	CBS 101239 ^T^	OQ429817	OQ430080	OQ454214	OQ471145	Hou et al. 2023
*Rosasphaeria moravica*	LMM ^T^	JF440985	-	JF440986	JF440987	Jaklitsch and Voglmayr 2012
*Roumegueriella rufula*	CBS 346.85	-	DQ518776	DQ522461	DQ522355	Spatafora et al. 2007
*R. rufula*	GJS 91-164	-	EF469082	EF469116	EF469070	Sung et al. 2007
*Sarocladium agarici*	CBS 113717 ^T^	OQ429828	OQ430089	OQ454227	OQ471158	Hou et al. 2023
*S. bacillisporum*	CBS 425.67 ^T^	NR_145039	MH870718	-	-	Hou et al. 2023
*S. dejongiae*	CBS 144929 ^T^	NR_161153	NG_067854	-	-	Hou et al. 2023
*S. gamsii*	CBS 707.73 ^T^	OQ429839	HG965063	OQ454238	OQ471169	Hou et al. 2023
*S. glaucum*	CBS 796.69 ^T^	OQ429841	HE608657	OQ451839	OQ471304	Hou et al. 2023
*S. implicatum*	CBS 959.72 ^T^	HG965023	MH878470	-	-	Hou et al. 2023
*S. kiliense*	CBS 122.29 ^T^	AJ621775	HQ232052	OQ454241	OQ471172	Hou et al. 2023
*S. ochraceum*	CBS 428.67 ^T^	OQ429846	HQ232070	OQ454245	OQ471176	Hou et al. 2023
*S. strictum*	CBS 346.70 ^T^	OQ429853	HQ232141	OQ454252	OQ471184	Hou et al. 2023
*S. subulatum*	CBS 217.35 ^T^	MH855652	NG_070566	-	-	Hou et al. 2023
*S. terricola*	CBS 243.59 ^T^	MH857853	MH869389	-	-	Hou et al. 2023
*Shimizuomyces paradoxus*	EFCC 6279 ^T^	JN049847	EF469084	EF469117	EF469071	Sung et al. 2007
*S. paradoxus*	EFCC 6564	-	EF469083	EF469118	EF469072	Sung et al. 2007
*Simplicillium lamellicola*	CBS 116.25	AJ292393	MH866307	DQ522462	DQ522356	Spatafora et al. 2007
*S. lanosoniveum*	CBS 101267	AJ292395	-	DQ522463	DQ522357	Spatafora et al. 2007
*S. lanosoniveum*	CBS 704.86	AJ292396	AF339553	DQ522464	DQ522358	Spatafora et al. 2007
*Sordaria fimicola*	AFTOL-ID 216 ^T^	DQ518178	-	-	DQ518175	James et al. 2006
*Sphaerostilbella aureonitens*	GJS74-87	FJ442633	HM466683	FJ442763	-	Schoch et al. 2012
*S. berkeleyana*	GJS82-274	-	U00756	-	AF543783	James et al. 2006
*S. chlorohalonata*	DAOM 235557	JN942888	JN938870	-	-	Schoch et al. 2012
*Stachybotrys eucylindrospora*	ATCC 18851	JN942887	JN938869	-	-	Schoch et al. 2012
*S. microspora*	CBS 186.79	-	-	DQ676580	DQ676604	Schoch et al. 2012
*Stephanonectria keithii*	GJS92-133 ^T^	-	AY489727	-	AY489622	Castlebury et al. 2004
*Tilachlidium brachiatum*	CBS 506.67	KM231839	HQ232177	KM232415	KM231976	Lombard et al. 2015
*T. brachiatum*	CBS 363.97	KM231838	KM231719	KM232414	KM231975	Lombard et al. 2015
*Tolypocladium inflatum*	SCALT1007-002^T^	KC963032	-	-	-	Ma et al. 2012
*Trichoderma aggressivum*	CBS100525	-	JN939837	JQ014130	-	Schoch et al. 2012
*T. viride*	GJS89-127	-	AY489726	-	AY489621	Castlebury et al. 2004
*Trichothecium crotocinigenum*	CBS 129.64 ^T^	OQ429885	OQ430137	-	OQ471217	Hou et al. 2023
*T. downum*	SICAUCC 23-0076^T^	PP060692	PP057975	-	-	Wang et al. 2025b
*T. downum*	SICAUCC 23-0155	PP844883	PP826169	-	-	Wang et al. 2025b
*T. hongkongense*	CBS 101444 ^T^	OQ429887	OQ430139	OQ454288	OQ471219	Hou et al. 2023
*T. hongkongense*	CBS 102186	OQ429886	OQ430138	OQ454287	OQ471218	Hou et al. 2023
*T. indicum*	CBS 123.78	OQ429889	OQ430141	-	OQ471221	Hou et al. 2023
*T. ovalisporum*	DAOM 186447 ^T^	NR_111321	-	-	-	Summerbell et al. 2011
*T. ovalisporum*	-	EU445372	-	-	EU445373	Summerbell et al. 2011
*T. roseum*	DUCC 502	JN937590	JX458860	-	-	Summerbell et al. 2011
*T. roseum*	DAOM 208997	JN942882	JN938864	-	-	Summerbell et al. 2011
*T. roseum*	CBS 566.50	MH856757	MH868278	-	-	Summerbell et al. 2011
*T. sinense*	DY05461^T^	PV688360	PV688368	PV705688	PV705696	This study
*T. sinense*	DY05462	PV688361	PV688369	PV705689	PV705697	This study
*T. sinense*	DY05591	PV688362	PV688370	PV705690	PV705698	This study
*T. sinense*	DY05592	PV688363	PV688371	PV705691	PV705699	This study
*T. sympodiale*	ATCC 36477 ^T^	-	NG_059884	-	-	Summerbell et al. 2011
*T. sympodiale*	CBS 227.76	MH860973	MH872742	-	-	Summerbell et al. 2011

Note. New strains or species are in bold type. “^T^” denotes ex-type. Abbreviations: ATCC, American Type Culture Collection, USA; BCC, BIOTEC Culture Collection, Klong Luang, Thailand; CBS, Westerdijk Fungal Biodiversity Institute (previously Centraal bureau voor Schimmelcultures), Utrecht, the Netherlands; CGMCC, China General Microbiological Culture Collection Center, China; DAOM, Department of Agriculture, Ottawa, Mycological Collections, Ottawa, Canada; EFCC, Entomopathogenic Fungal Culture Collection, Chuncheon, Korea; GMBC, Guizhou Medical University Culture Collection, Guiyang, China; NHJ, Nigel Hywel-Jones personal collection; OSC, Oregon State University Herbarium, Corvallis, OR; YHH, Yunnan Herbal Herbarium, Kunming, China.

### ﻿Sequence alignments and phylogenetic analyses

DNASTAR™ Lasergene (v.6.0) was used to edit DNA sequences in this study. The ITS, LSU, RPB2 and *tef*-1α sequences for this analysis were downloaded from GenBank based on recent, related studies (e.g. Hou et al. 2023; Lin et al. 2025; Zhao et al. 2025; Wang et al. 2025b), and other sequences were selected based on BLASTn similarity searches. All the sequences were aligned by MAFFT v.7.037b (Katoh and Standley 2013) and alignments edited with MEGAv. 6 (Tamura et al. 2013).

We carried out four phylogenetic analyses to confirm the placement of the strains in different taxonomic hierarchies.

- Analyses 1: Analyses based on ITS, LSU, *RPB2* and *tef*-1α gene regions to confirm the familialplacement of the new strains.
- Analysis 2: Analyses based on ITS, LSU, *RPB2* and *tef*-1α gene regions to show the placement of new strains in *Ovicilliums. str.*
- Analysis 3: Analyses based on ITS, LSU, *RPB2* and *tef*-1α gene regions to show the placement of new strains in *Conoideocrellas. str.*
- Analysis 4: Analyses based on ITS, LSU, *RPB2* and *tef*-1α gene regions to show the placement of new strains in *Trichotheciums. str.*

The combined datasets of ITS, LSU, *RPB2* and *tef*-1α gene regions were obtained using SequenceMatrixv.1.7.8 (Vaidya et al. 2011). The model for each data partition was selected for Bayesian analysis by ModelFinder (Kalyaanamoorthy et al. 2017) in the PhyloSuite v. 1.2.2 software (Zhang et al. 2020).

The datasets 1–4 were analyzed using Bayesian inference (BI) and maximum likelihood (ML) methods, respectively. For BI, a Markov chain Monte Carlo (MCMC) algorithm was used to generate phylogenetic trees with Bayesian probabilities for the combined sequence datasets using MrBayes v.3.2 (Ronquist et al. 2012). The Bayesian analysis resulted in 20,001 trees after 10,000,000 generations. The first 4,000 trees, representing the burn-in phase of the analysis, were discarded, while the remaining 16,001 trees were used to calculate posterior probabilities in the majority rule consensus tree. After the analysis was finished, each run was examined to see if it was greater than 200 using the program Tracer v.1.5 (Drummond and Rambaut 2007) to determine burn-in and confirm that both runs had converged. The ML analyses were constructed with IQ-TREE v. 2.0 (Nguyen et al. 2015; Trifinopoulos et al. 2016), using the ML+rapid bootstrap setting with 1,000 replicates and an automatic selection of the model according to BIC.

## ﻿Results

### ﻿Phylogenetic analyses

Analysis 1

**Figure 1.**
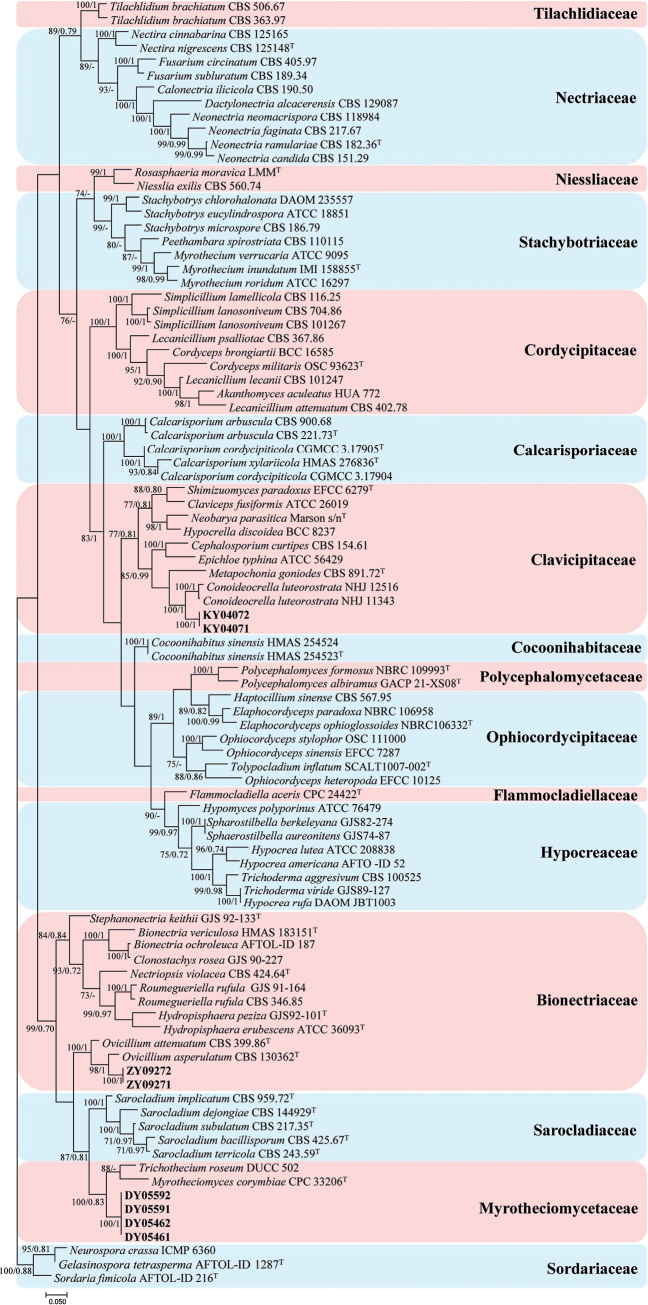
Phylogram retrieved from IQ-TREE to confirm the familial placements of the new strains using the combined dataset of ITS, LSU, *RPB2* and *tef*-1α gene regions. The statistical values are provided at nodes as ML/PP (ML value above 70% and BI value above 0.70). The tree is rooted with *Gelasinospora tetrasperma* (AFTOL-ID 1287), *Neurospora crassa* (ICMP 6360) and *Sordaria fimicola* (AFTOL-ID 216). Ex-types and new strains are indicated by the superscript “T” and in bold, respectively.

The familial placements of the new strains are confirmed in this analysis (Fig. 1). *Gelasinospora tetrasperma* Dowding (AFTOL-ID 1287), *Neurospora crassa* Shear & B.O. Dodge (ICMP 6360) and *Sordariafimicola* (Roberge ex Desm.) Ces. & De Not. (AFTOL-ID 216) were used as the outgroup taxa in the analysis. The dataset included 79 taxa, and consisted of 3,235 (ITS, 686; LSU, 842; *RPB2*, 882, and *tef*-1α, 825) characters with alignment gaps.

The selected model for the ML analysis was TIM2+F+G4. The final value of the highest scoring tree was –55,119.994, which was obtained from the ML analysis of the dataset. The parameters of the GTR model used to analyze the dataset were estimated based on the following frequencies: A = 0.236, C = 0.272, G = 0.276, T = 0.215; substitution rates AC = 1.27070, AG = 2.13314, AT = 1.27070, CG = 1.00000, CT = 5.12614 and GT = 1.00000, as well as the gamma distribution shape parameter α = 0.468. The selected model of the dataset for BI analysis was GTR+F+I+G4 (ITS), GTR+F+G4 (LSU, *tef*-1α) and SYM+I+G4 (*RPB2*). The phylogenetic tree (Fig. 1) constructed using ML and BI analyses was largely congruent and strongly supported in most branches.

Strains KY04071 and KY04072 clustered sister to *Conoideocrella luteorostrata* (Zimm.) D. Johnson et al. (NHJ 11343 and NHJ 12516) and formed a stable clade in the family Clavicipitaceae.

Strains DY05461, DY05462, DY05591 and DY05592 clustered sister to *Trichothecium roseum* (Pers.) Link (DUCC 502) and *Myrotheciomyces corymbiae* Crous (CPC 33206) in the family Myrotheciomycetaceae.

Strains ZY09271 and ZY09272 clustered sister to *Ovicillium asperulatum* (Giraldo et al.) L.W. Hou et al. (CBS 130362) and *O. attenuatum* Zare & W. Gams (CBS 399.86) in the family Bionectriaceae.

#### ﻿Analysis 2

**Figure 2.**
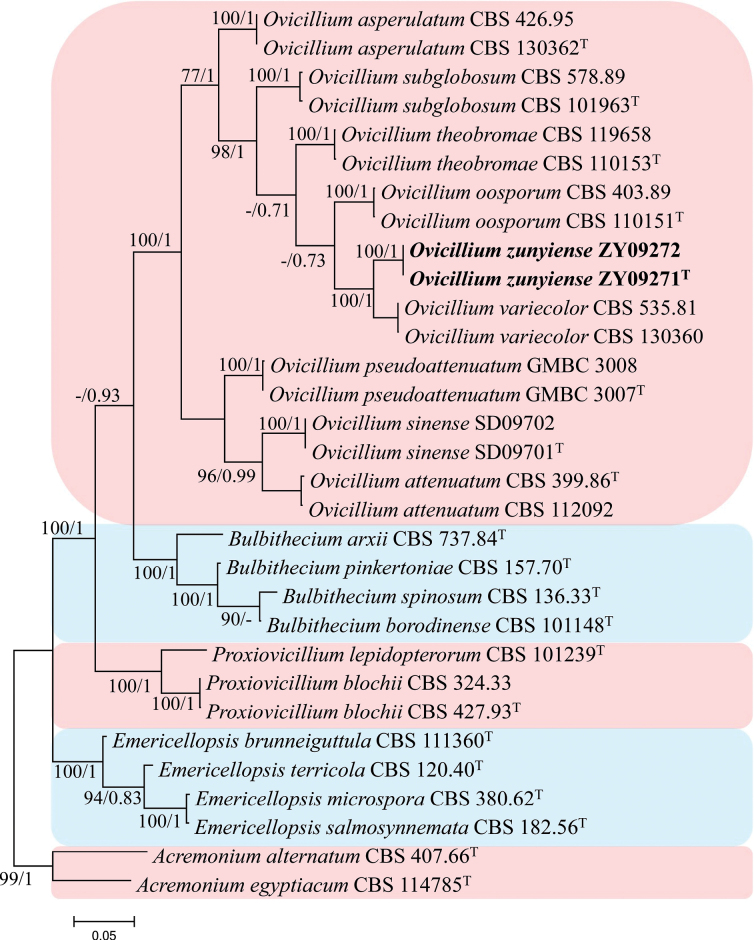
Phylogram retrieved from IQ-TREE for establishing the new species in the genus *Ovicilliums. str.* using the combined dataset of ITS, LSU, *RPB2* and *tef*-1α gene regions. The statistical values are provided at nodes as ML/PP (ML value above 70% and BI value above 0.70). The tree is rooted with *Acremonium alternatum* (CBS 407.66) and *A. egyptiacum* (CBS 114785). Ex-types and new strains are indicated by the superscript “T” and in bold, respectively.

Phylogenetic trees were generated in analysis 2for establishing the new species in the genus *Ovicillium* (Fig. 2). *Acremonium alternatum* Link (CBS 407.66) and *A. egyptiacum* (J.F.H. Beyma) W. Gams (CBS 114785) were used as the outgroup taxa in the analysis. The dataset included 20 taxa and consisted of 2,872 (ITS, 517; LSU, 777; *RPB2*, 767, and *tef*-1α, 811) characters with alignment gaps.

The selected model for the ML analysis was TN+F+I+G4. The final value of the highest scoring tree was –12,780.840, which was obtained from the ML analysis of the dataset. The parameters of the GTR model used to analyze the dataset were estimated based on the following frequencies: A = 0.235, C = 0.276, G = 0.268, T = 0.220; substitution rates AC = 1.00000, AG = 2.52675, AT = 1.00000, CG = 1.00000, CT = 5.98208 and GT = 1.00000, as well as the gamma distribution shape parameter α = 0.800. The selected model of the dataset for BI analysis was GTR+F+I+G4 (ITS, LSU, *tef*-1α) and SYM+I+G4 (*RPB2*). The phylogenetic tree (Fig. 2) constructed using ML and BI analyses was largely congruent and strongly supported in most branches. Strains ZY09271 and ZY09272 formed an independent clade with high statistical support (100% ML/1 PP) and were clustered with *Ovicillium oosporum* Zare & W. Gams, *O. subglobosum* Zare & W. Gams, *O. theobromae* Lin Zhao bis & Crous, *O. variecolor* (Giraldo et al.) L.W. Hou et al. in a clade with high statistical support in ML and BI analysis (98% ML/1 PP).

#### ﻿Analysis 3

**Figure 3.**
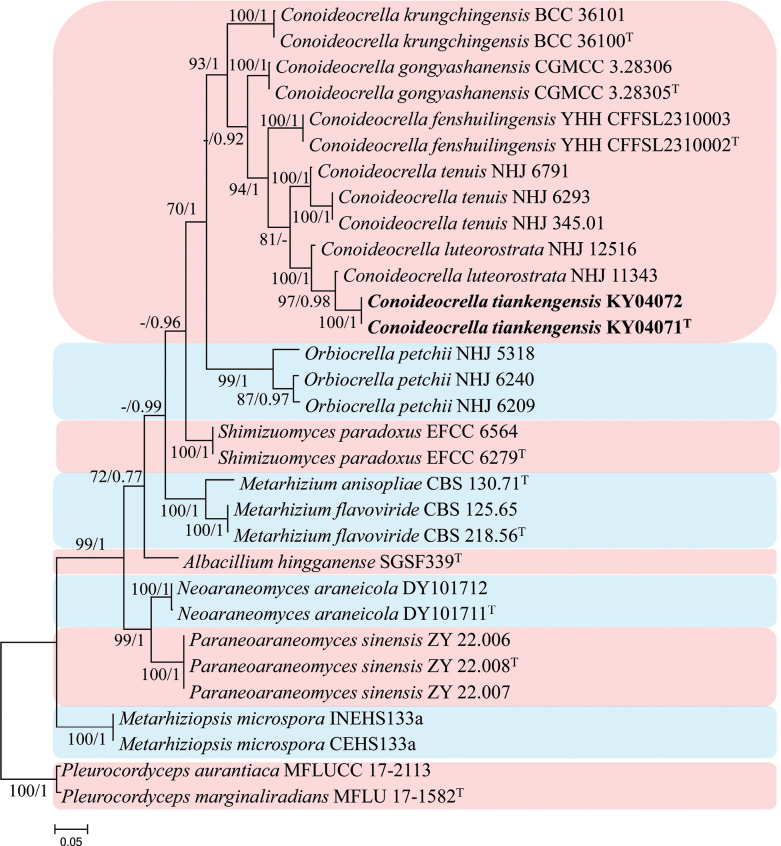
Phylogram retrieved from IQ-TREE of the placement of new strains in *Conoideocrellas. str.* using the combined dataset of ITS, LSU, *RPB2* and *tef*-1α gene regions. The statistical values are provided at nodes as ML/PP (ML value above 70% and BI value above 0.70). The tree is rooted with *Pleurocordyceps aurantiaca* (MFLUCC 17-2113) and *P. marginaliradians* (MFLU 17-1582). Ex-types and new strains are indicated by the superscript “T” and in bold, respectively.

Phylogenetic trees were generated in analysis 3 for establishing the new species in the genus *Conoideocrella* (Fig. 3). *Pleurocordyceps aurantiaca* (Y.P. Xiao et al.) Y.H. Wang et al. (MFLUCC 17-2113) and *P. marginaliradians* (Y.P. Xiao et al.) Y.H. Wang et al. (MFLU 17-1582) were used as the outgroup taxa in the analysis. The dataset included 20 taxa and consisted of 3,279 (ITS, 642; LSU, 819; *RPB2*, 917, and *tef*-1α, 901) characters with alignment gaps.

The selected model for the ML analysis was TN+F+I+G4. The final value of the highest scoring tree was –14,463.202, which was obtained from the ML analysis of the dataset. The parameters of the GTR model used to analyze the dataset were estimated based on the following frequencies: A = 0.236, C = 0.271, G = 0.276, T = 0.217; substitution rates AC = 1.00000, AG = 2.83420, AT = 1.00000, CG = 1.00000, CT = 8.61744 and GT = 1.00000, as well as the gamma distribution shape parameter α = 0.591. The selected model of the dataset for BI analysis was HKY+F+G4 (ITS), GTR+F+I+G4 (LSU, *tef*-1α) and SYM+I+G4 (*RPB2*). The phylogenetic tree (Fig. 3) constructed using ML and BI analyses was largely congruent and strongly supported in most branches. Strains KY04071 and KY04072 formed an independent clade with high statistical support (100% ML/1 PP) and clustered with *Conoideocrella luteorostrata* in a clade with high statistical support in ML and BI analysis (100% ML/1 PP).

#### ﻿Analysis 4

**Figure 4.**
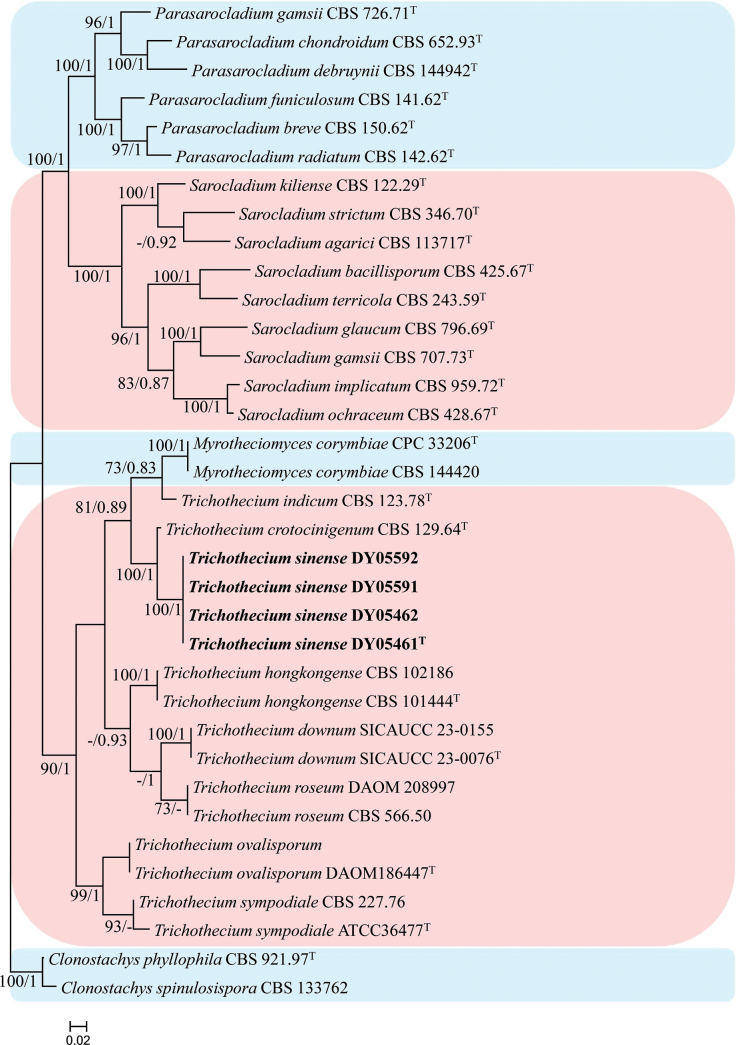
Phylogram retrieved from IQ-TREE of the placement of new strains in *Trichotheciums. str.* using the combined dataset of ITS, LSU, *RPB2* and *tef*-1α gene regions. The statistical values are provided at nodes as ML/PP (ML value above 70% and BI value above 0.70). The tree is rooted with *Clonostachys phyllophila* (CBS 921.97) and *Clonostachys spinulosispora* (CBS 133762). Ex-types, new strains are indicated by the superscript “T” and in bold, respectively.

Phylogenetic trees were generated in analysis 4 for establishing the new species in the genus *Trichothecium* (Fig. 4). *Clonostachys phyllophila* Schroers (CBS 921.97) and *Clonostachys spinulosispora* Lechat & J. Fourn. (CBS 133762) were used as the outgroup taxa in the analysis. The dataset included 25 taxa, and consisted of 2,994 (ITS, 569; LSU, 779; *RPB2*, 780, and *tef*-1α, 866) characters with alignment gaps.

The selected model for the ML analysis was TIM2+F+I+G4. The final value of the highest scoring tree was –17,969.800, which was obtained from the ML analysis of the dataset. The parameters of the GTR model used to analyze the dataset were estimated based on the following frequencies: A = 0.228, C = 0.283, G = 0.280, T = 0.209; substitution rates AC = 1.39668, AG = 2.51979, AT = 1.39668, CG = 1.00000, CT = 6.81690 and GT = 1.00000, as well as the gamma distribution shape parameter α = 0.586. The selected model of the dataset for BI analysis was GTR+F+I+G4 (ITS, LSU, *tef*-1α) and SYM+I+G4 (*RPB2*). The phylogenetic tree (Fig. 4) constructed using ML and BI analyses was largely congruent and strongly supported in most branches. Strains DY05461, DY05462, DY05591 and DY05592 formed an independent clade with high statistical support (100% ML/1 PP) and were clustered with *Trichothecium crotocinigenum* (Schol-Schwarz) Summerb. et al. in a clade with high statistical support in ML and BI analysis (99% ML/1 PP).

In addition, the type strain of *Myrotheciomyces corymbiae* Crous (CPC 33206), the type species of *Myrotheciomyces* and another strain of the same species (i.e. CBS144420) (Crous et al. 2018) reside in the *Trichotheciums. str.* clade. This placement agrees with Hou et al. (2023), hence, we propose to regard the younger, asexual typified name as a synonym of the older, asexual typified name, i.e. *Trichothecium* over *Myrotheciomyces*.

### ﻿Taxonomy

**Bionectriaceae Samuels & Rossman, Stud. Mycol. 42: 15, 1999**

***Ovicillium* Zare & W. Gams, Mycol. Progr. 15: 1020, 2016**

#### ﻿ Ovicillium zunyiense

Taxon classificationFungiHypocrealesBionectriaceae

W.H. Chen, Y.F. Han, J.D. Liang & J.H. Zhao sp. nov.

1F975BCF-52A7-52F4-95CB-441F5A8098C2

859496

Fig. 5

##### Etymology.

Referring to its location, Zunyi City, where the fungus was first discovered.

##### Type.

China • Guizhou Province, Zunyi City, Honghuagang District, Dabanshui National Forest Park (27°46'35.904"N, 106°48'30.89"E). On a dead larva (Lepidoptera), on the leaf litter, 2 September 2023, Wanhao Chen, GZAC ZY0927, holotype; ZY09271, ex-type.

**Figure 5.**
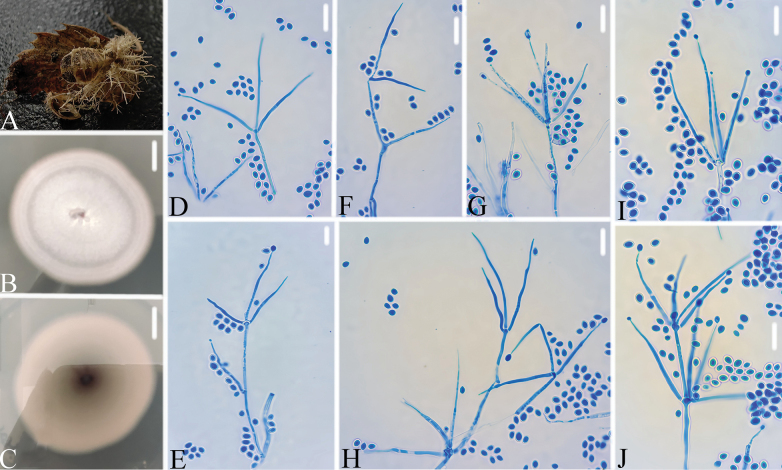
*Ovicillium zunyiense* A. Infected larva; B, C. PDA culture plate showing the top (B) and reverse (C) sides of the colony; D–J. Phialides and conidia. Scale bars: 10 mm (B, C) and 10 μm (D–J).

##### Diagnosis.

Differs from *Ovicillium oosporum* by its shorter conidiophore, smaller conidia and its insect substrate. Differs from *O. subglobosum* by its shorter phialides, smaller ovoid to subglobose conidia and insect substrate. Differs from *O. theobromae* by its shorter conidiophores, shorter phialides and insect substrate. Differs from *O. variecolor* by its shorter conidiophores, shorter phialides, absence of sessile conidia and insect substrate.

##### Description.

Colonies on PDA, attaining a diameter of 42–45 mm after 14 days at 25 °C, grayish-white to light brown, consisting of a basal felt, floccose hyphal overgrowth; reverse light brown to brown. Hyphae septate, hyaline, smooth-walled, 1.6–1.7 μm wide. Conidiophores hyaline, smooth-walled, with single phialide or whorls of 2–4 phialides or verticillium-like from hyphae directly, 17.4–26.2 × 2.3–3.0 μm (x̄= 20.8 × 2.6 μm, n = 30). Phialides cylindrical, somewhat inflated base, 21.6–33.3 × 1.2–2.6 μm (x̄= 28.2 × 1.7 μm, n = 30), tapering to a thin neck. Conidia hyaline, smooth-walled, ovoid to subglobose, 2.3–3.7 × 1.7–2.6 μm (x̄= 2.7 × 2.1 μm, n = 30). Sexual state not observed.

##### Host.

Larva (Lepidoptera).

##### Habitat.

Near the road, located on the leaf litter.

##### Additional strain examined.

China • Guizhou Province, Zunyi City, Honghuagang District, Dabanshui National Forest Park (27°46'35.904"N, 106°48'30.89"E). On a dead larva (Lepidoptera),on the leaf litter, 2 September 2023, Wanhao Chen, ZY09272 (living culture).

##### Remarks.

Based on BLASTn results, strains ZY09271 and ZY09272 were identified as members of *Ovicilliums. str.*, and the phylogenetic analysis of the combined datasets 1 and 2 (Figs 1, 2). It clustered into an independent clade with a close relationship with *Ovicillium oosporum*, *O. subglobosum*, *O. theobromae* and *O. variecolor* with a high support value (98% ML/1 PP). Compared with the typical characteristics, *Ovicillium zunyiense* can be distinguished from *O. oosporum* by its shorter conidiophores (17.4–26.2 × 2.3–3.0 μm vs. 20–50 × 1.2–2.2 μm), smaller conidia (2.3–3.7 × 1.7–2.6 μm vs. 4–6 × 2.5–4.0 μm) and its substrates (insect vs. plant). *Ovicillium zunyiense* can be distinguished from *O. subglobosum* by its short phialides (21.6–33.3 × 1.2–2.6 μm vs. 25–55 × 1.5–2.2 μm), smaller ovoid to subglobose conidia (2.3–3.7 × 1.7–2.6 μm vs. 3.5–5.5 × 3.5–4.5 μm) and its substrates (insect vs. soil). *Ovicillium zunyiense* can be distinguished from *O. theobromae* by its short conidiophores (17.4–26.2 × 2.3–3.0 μm vs. 460 × 1.5–3.2 μm), short phialides (21.6–33.3 × 1.2–2.6 μm vs. 28.4–65.5 × 1.2–1.8 μm) and its substrates (insect vs. plant). *Ovicillium zunyiense* can be distinguished from *O. variecolor* by its short conidiophores (17.4–26.2 × 2.3–3.0 μm vs. 290 μm long), short phialides (21.6–33.3 × 1.2–2.6 μm vs. 18–95 × 1–2 μm), absence of sessile conidia and its substrates (insect vs. soil). Thus, the morphological characteristics and molecular phylogenetic results support to establishment of *O. zunyiense*as a new species.

### ﻿Taxonomic key to distinguish the species of *Ovicillium*

**Table:** 

1	Chlamydospores present	**2**
–	Chlamydospores absent	**3**
2	Chlamydospores abundant, Conidia globose	***Ovicillium asperulatum***
–	Scarce chlamydospores may be present, Conidia subglobose, oval to broadly oval	***Ovicillium oosporum***
3	The sessile conidia absent	**4**
–	The sessile conidia present	***Ovicillium variecolor***
4	Conidia oval, subglobose or globose	**5**
–	Conidia ellipsoidal to cylindrical	***Ovicillium pseudoattenuatum***
5	Conidiophore smooth	**6**
–	Conidiophore near the base roughened	***Ovicillium attenuatum***
6	Soil or plant substrates	**7**
–	Insect substrates	**8**
7	Soil substrates, conidia subglobose, 3.5–5.5 × 3.5–4.5 μm	***Ovicillium subglobosum***
–	Plant substrates, conidia subglobose or ellipsoid, (2.8–)3.0–3.7(–4.4) × (2.2–)2.3–2.9(–3.1) μm	***Ovicillium theobromae***
8	Conidia globose to ovoid, 2.1–2.9 × 1.1–1.7 μm	***Ovicillium sinense***
–	Conidia ovoid to subglobose, 2.3–3.7 × 1.7–2.6 μm	***Ovicillium zunyiense***

#### ﻿Clavicipitaceae Rogerson, Mycologia 62(5): 900, 1970

***Conoideocrella* D. Johnson, G.H. Sung, Hywel-Jones & Spatafora, Mycol. Res. 113(3): 286, 2009**

##### ﻿ Conoideocrella tiankengensis

Taxon classificationFungiHypocrealesBionectriaceae

W.H. Chen, Y.F. Han, J.D. Liang & J.H. Zhao sp. nov.

017EFA6B-2088-5863-AB71-BE30D258DEB2

859499

Fig. 6

###### Etymology.

Referring to its location, Monkey-Ear Tiankeng, where the fungus was first discovered.

**Figure 6.**
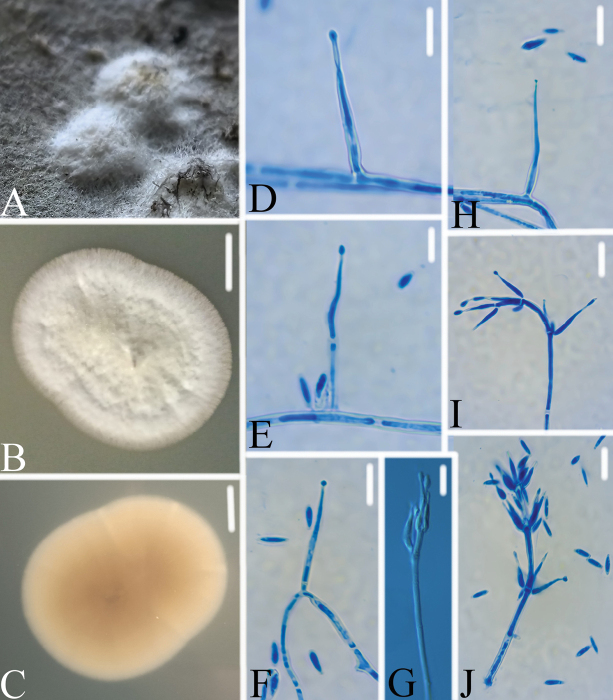
*Conoideocrella tiankengensis* A. Infected scale insect; B, C. PDA culture plate showing the top (B) and reverse (C) sides of the colony; D–J. Conidiogenous structures and conidia. Scale bars: 10 mm (B, C) and 10 μm (D–J).

###### Type.

China • Guizhou Province, Guiyang City, Monkey-Ear Tiankeng (27°5'12.138'’ N, 107°0'48.42'’ E). On a dead scale insect (Coccoidea),on the leaf, 6 April 2024, Wanhao Chen, GZAC KY0407, holotype; KY04071, ex-type.

###### Diagnosis.

Differs from *Conoideocrella luteorostrata* by its shorter and hyaline conidiophore, two types of phialides and fusiform to filiform conidia.

###### Description.

Colonies grow slowly on PDA at 25 ^◦^C, attaining a diam. of 26–39 mm in 14 days, white to cream-white mycelium at first, turning pale yellow with age. Colonies loose on the edge and compact in the middle. Hyphae smooth, septate, hyaline, 1.7–2.4 μm wide. Conidiophores hyaline, smooth-walled, with single phialide or whorls of 2–4 phialides or verticillium-like from hyphae directly, 13.6–23.2 × 1.6–2.6 μm (x̄= 17.2 × 2.0 μm, n = 30). Two types of conidiogenous structures were present. Hirsutella-like asexual state arises from hyphae, conidiogenous structures with slender base tapering more or less evenly to a neck, hyaline, produced directly on the hyphae, 15.1–27.1 × 1.6–1.8 μm (x̄= 21.4 × 1.7 μm, n = 30). Isaria-like conidiogenous structures also arises from hyphae, cylindrical to ellipsoidal, somewhat inflated base, tapering to a thin neck, 9.8–13.5 × 1.4–1.8 μm (x̄= 10.8 × 1.7 μm, n = 30). Conidia hyaline, smooth, fusiform to filiform, forming short divergent and basipetal chains, 5.3–6.7 × 1.6–2.2 μm (x̄= 5.9 × 1.8 μm, n = 30).

###### Host.

Scale insect (Coccoidea).

###### Habitat.

Near the road, located on the leaf.

###### Additional strain examined.

China • Guizhou Province, Guiyang City, Monkey-Ear Tiankeng (27°5'12.138'’ N, 107°0'48.42'’ E). On a dead scale insect (Coccoidea), on the leaf, 6 April 2024, Wanhao Chen, KY04072 (living culture).

###### Remarks.

*Conoideocrella tiankengensis* was identified as *Conoideocrella*, based on the BLASTn result in NCBI and the phylogenetic analysis of the combined datasets 1 and 3 (Figs 1, 3). It clustered into an independent clade with a close relationship with *C. luteorostrata* with high statistical values (100% ML/1 PP). Compared with the typical characteristics, *C. tiankengensis* can be distinguished from *C. luteorostrata* by its shorter and hyaline conidiophore (13.6–23.2 × 1.6–2.6 μm vs. 150–240 × 2.0–3.0 μm), two types of phialides and fusiform to filiform conidia (Hywel-Jones 1993). Thus, the morphological characteristics and molecular phylogenetic results support *C. tiankengensis* as a new species.

### ﻿Taxonomic key to distinguish the species of *Conoideocrella*

**Table:** 

1	The sexual morphabsent	**2**
–	The sexual morph present	**3**
2	Spider host, Hirsutella-like conidiogenous structure, 12.7–89.9 × 0.4–1.3 μm	***Conoideocrella gongyashanensis***
–	Scale insect host, Hirsutella-like and isaria-like conidiogenous structure, 15.1–27.1 × 1.6–1.8 μm and 9.8–13.5 × 1.4–1.8 μm, respectively	***Conoideocrella tiankengensis***
3	Stromata flattened pulvinate to discoid, planar, pulvinate, almost planar	**4**
–	Stromata scutate or hemi-globose	***Conoideocrella fenshuilingensis***
4	Perithecia < 600 μm long	5
–	Perithecia > 600 μm long	***Conoideocrella luteorostrata***
5	Stromata pale yellow, orange to reddish brown; Asci < 180 μm long; Conidia 8–15 × 2–4 μm	***Conoideocrella krungchingensis***
–	Stromata white to orangish-pink; Asci > 180 μm long; Conidia 6.1–12.5 × 1.3–2.3 μm	***Conoideocrella tenuis***

#### ﻿Myrotheciomycetaceae Crous, Persoonia 40: 351, 2018

##### ﻿ Trichothecium

Taxon classificationFungiHypocrealesMyrotheciomycetaceae

Link, Mag. Gesell. naturf. Freunde, Berlin 3(1-2): 18, 1809

2A2908FC-8F51-5BBF-ABB1-F0509EA65772

=Myrotheciomyces Crous, Persoonia 40: 351, 2018; MycoBank no.: 825409

###### Note.

The family Myrotheciomycetaceae was introduced by Crous et al. (2018) with four genera, *Emericellopsis* J.F.H. Beyma, *Leucosphaerina* Arx, *Myrotheciomyces* Crous and *Trichothecium* Link. Bao et al. (2023) exclude *Emericellopsis* from Myrotheciomycetaceae based on the phylogenetic analysis and showed that the type strain of *Myrotheciomyces corymbiae* (CPC 33206) is accommodated in *Trichotheciums. str.* In the present study, the type strain of *Myrotheciomyces corymbiae* clustered in the *Trichothecium* clade (Fig. 4). Thus, we propose to synonymize *Myrotheciomyces* with *Trichothecium* as the latter is the older generic epithet.

##### ﻿ Trichothecium sinense

Taxon classificationFungiHypocrealesMyrotheciomycetaceae

W.H. Chen, Y.F. Han, J.D. Liang & J.H. Zhao sp. nov.

63D9201E-C3F2-5C2F-89B4-D026E3EC23C3

859500

Fig. 7

###### Etymology.

Referring to its location, China, where the fungus was first discovered.

**Figure 7.**
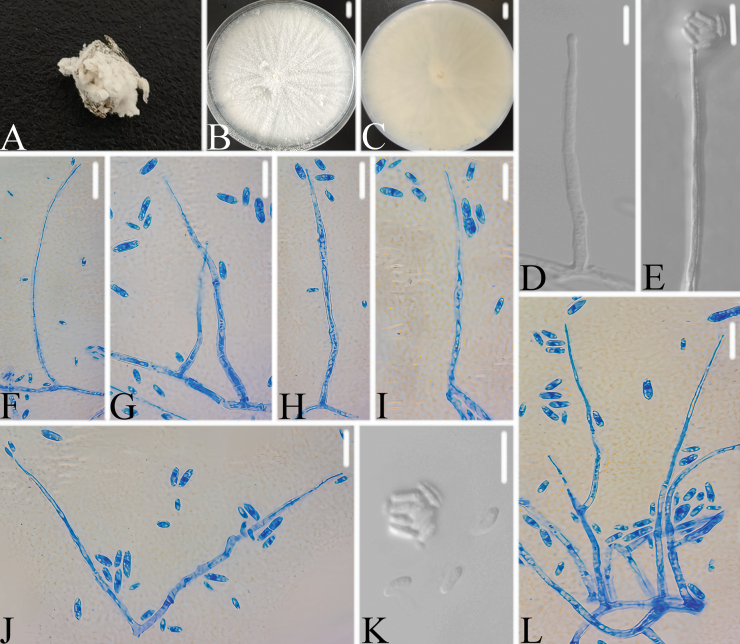
*Trichothecium inense* A. Infected spider; B, C. PDA culture plate showing the top (B) and reverse (C) sides of the colony; D–L. Phialides and conidia. Scale bars: 10 mm (B, C) and 10 μm (D–L).

###### Type.

China • Guizhou Province, Qiannan Buyi and Miao Autonomous Prefecture, Duyun City, Mayao River Valley (26°21'24.71"N, 107°22'48.22"E). On a dead spider (Araneae),on or under rocks, 1 May 2022, Wanhao Chen, GZAC DY0546, holotype;DY05461, ex-type.

###### Diagnosis.

Differs from *Trichothecium crotocinigenum* by its shorter phialides, larger conidia and spider host.

###### Description.

Colonies on PDA, attaining a diameter of 86–90 mm after 14 days at 25 °C, white, consisting of a basal felt, floccose hyphal overgrowth; reverse light yellow. Conidiophores solitary, (sub-)erect, arising directly from submerged or superficial hyphae, 14.3–23.1 × 1.4–2.6 μm (x̄= 17.7 × 1.8 μm, n = 30). Phialides lateral or terminal, cylindrical, occasionally swollen in the lower part, hyaline, thick-, smooth-walled, 32.8–55.1 × 1.9–3.0 μm (x̄= 46.9 × 2.6 μm, n = 30). Conidia aseptate, cylindrical, oblong or ovoid, rounded at both ends, hyaline, thin-, smooth-walled, 4.8–5.8 × 1.2–2.9 μm (x̄= 5.5 × 2.4 μm, n = 30), arranged in slimy heads. Chlamydospores not observed. Sexual morph not observed.

###### Host.

Spider (Araneae).

###### Habitat.

Near the road, located on or under rocks.

###### Additional material examined.

China • Guizhou Province, Qiannan Buyi and Miao Autonomous Prefecture, Duyun City, Mayao River Valley (26°21'24.71"N, 107°22'48.22"E). On a dead spider (Araneae),on or under rocks, 1 May 2022, Wanhao Chen, DY05462 (living culture); GZAC DY0559 (specimen), DY05591, DY05592 (living culture).

###### Remarks.

*Trichothecium sinense*was identified as *Trichothecium*, based on the BLASTn result in NCBI and the phylogenetic analysis of the combined datasets 1and 4 (Figs 1, 4). It clustered into an independent clade with a close relationship with *Trichothecium crotocinigenum* with high statistical values (99% ML/1 PP). Compared with the typical microscopic characteristics, *Trichothecium sinense* can be distinguished from *T. crotocinigenum* by its shorter phialides (32.8–55.1 × 1.9–3.0 μm vs. 168 μm long), larger conidia [4.8–5.8 × 1.2–2.9 μm vs. 3–8(–11) × 2–3 μm] and its substrates (mushroom vs. spider). Thus, the morphological characteristics and molecular phylogenetic results support *T. sinense* as a new species.

### ﻿New combination

#### ﻿ Trichothecium corymbiae

Taxon classificationFungiHypocrealesMyrotheciomycetaceae

(Crous) W.H. Chen &Wijayaw. comb. nov.

51AEDE40-1F26-5931-98A3-64285DD743F1

859678

= Myrotheciomyces scorymbiae Crous, Persoonia 40: 351, 2018.

##### Note.

We proposed to treat *Myrotheciomyces* as a synonym of *Trichothecium* based on the phylogenetic analyses (Figs 1, 4) and therefore transferred *Myrotheciomyces corymbiae* to the genus *Trichothecium* as *Trichothecium corymbiae*.

## ﻿Discussion

Guizhou Province, as a typical karst region, exhibits exceptional habitat diversity, including plains, mountains, hills, caves, valleys, and forests. Hypocrealean fungi in this region have been extensively studied and current research reveal a significant concentration of Clavicipitaceae, Cordycipitaceae, and Ophiocordycipitaceae (Chen et al. 2022b, 2024d, 2025; Zhang et al. 2023; Bu et al. 2025; Xu et al. 2025). At the same time, other families remain understudied or poorly known. Moreover, these investigations have been predominantly limited to soil and forest ecosystems. Previous studies have revealed a remarkably high species diversity of Hypocrealean fungi in karst tiankengs and valley habitats, warranting further scientific investigation (Chen et al. 2022b, 2023, 2024d; Jiang et al. 2025). Recent taxonomic assessments highlight the need for expanded biodiversity surveys in karst ecosystems, particularly in under-characterized microhabitats (Wijayawardene et al. 2021).

In the present study, three new species, *Conoideocrella tiankengensis*, *Ovicillium zunyiense* and *Trichothecium sinense* were collected from Tiankeng, a karst forest and valley; the three species belong to the families Clavicipitaceae, Bionectriaceae and Myrotheciomycetaceae respectively. All three aforementioned fungal species demonstrate obligate associations with arthropod hosts. Notably, species in the genus *Conoideocrella* D. Johnson, G.H. Sung, Hywel-Jones & Spatafora all parasitize scale insects, with the exception of *C. gongyashanensis* (L.B. Lin & J.Z. Qiu), which parasitizes spiders (Hywel-Jones 1993; Mongkolsamrit et al. 2016; Wang et al. 2024; Lin et al. 2025). *Conoideocrella* species had only been documented in Yunnan and Fujian Province (Wang et al. 2024; Lin et al. 2025), and the new species was introduced from a karst habitat for the first time.

The genus *Trichothecium* Link was introduced with the type species *Trichothecium roseum* (Pers.) Link. Summerbell et al. (2011) revised *Trichothecium* and restricted this genus into five species: *Trichothecium crotocinigenum*, *T. indicum* (Arx, Mukerji & N. Singh) Summerbell, Seifert, & Schroers, *T. ovalisporum* (Seifert & Rehner) Seifert & Rehner, *T. roseum* and *T. sympodiale* Summerbell, Seifert, & Schroers. Hou et al. (2023) introduced a new species *Trichothecium hongkongense* L.W. Hou, L. Cai & Crous. These species have been mainly reported from covers manure, living leaves, mushroom, cereal silage and leaf-cutter bee (MycoBank, https://www.mycobank.org/). In this study, anarthropod-associated species, *T. sinense*, is introduced for the first time. Further research is needed to elucidate the environmental adaptation mechanisms in these species. Moreover, a new species in the family Myrotheciomycetaceae was introduced from Guizhou karst habitat for the first time.

The genus *Ovicillium* Zare & W. Gams was introduced with the type species, *O. attenuatum* Zare & W. Gams (Zare and Gams 2016). Chen et al. (2024a) summarized that the genus *Ovicillium* consists of five species and reported a new species *Ovicillium sinense* Wan H. Chen, Y.F. Han & J.D. Liang. Two new species *Ovicillium pseudoattenuatum* Y. Wang & D.X. Tang and *O. theobromae* Lin Zhao & Crous, were isolated from soil and plant by Wang et al. (2025c) and Zhao et al. (2025). Among these, only two species, *Ovicillium sinense* and *O. zunyiense* were arthropod-associated species. Whether this correlates with their ambient environment warrants further investigation.

Fungi in the order Hypocreales exhibit an evolutionary progression of trophic modes, transitioning from plant-based nutrition (including both living tissues and plant debris) to animal hosts (particularly insects), and ultimately to fungal substrates (Spatafora et al. 2007). This stepwise adaptation reflects an ecological optimization strategy for acquiring optimal nutrient resources (Behie et al. 2013; Behie and Bidochka 2014; Moonjely et al. 2016; Vidhate et al. 2023). Our study introduced two arthropod-associated species, *Ovicillium zunyiense* and *Trichothecium sinense*, which are seldom reported in the genera *Ovicillium* and *Trichothecium*. How environmental factors drive host-specific adaptations in these fungi is worthy of further research.

## Supplementary Material

XML Treatment for Ovicillium zunyiense

XML Treatment for Conoideocrella tiankengensis

XML Treatment for Trichothecium

XML Treatment for Trichothecium sinense

XML Treatment for Trichothecium corymbiae

## ﻿Additional information

### Use of AI

No use of AI was reported.

### Author ORCIDs

Wan-Hao Chen https://orcid.org/0000-0001-7240-6841

Jian-Dong Liang https://orcid.org/0000-0002-3939-3900

Nalin N. Wijayawardene https://orcid.org/0000-0003-0522-5498

Yan-Feng Han https://orcid.org/0000-0002-8646-3975

### Data availability

All of the data that support the findings of this study are available in the main text or Supplementary Information.

## Supplementary materials

Supplementary material 1 List of recent studies reported new arthropods-associated fungi in nine families Data type docx https://binary.pensoft.net/file/1441948This dataset is made available under the Open Database License (http://opendatacommons.org/licenses/odbl/1.0/). The Open Database License (ODbL) is a license agreement intended to allow users to freely share, modify, and use this Dataset while maintaining this same freedom for others, provided that the original source and author(s) are credited.Wan-Hao Chen, Hui-Lin Shu, Dan Li, Jian-Dong Liang, Xiu-Xiu Ren, Nalin N. Wijayawardene, Yan-Feng Han, Jie-Hong Zhao

Supplementary material 2 Aligement of ITS for dataset 1 Data type fas https://binary.pensoft.net/file/1441949This dataset is made available under the Open Database License (http://opendatacommons.org/licenses/odbl/1.0/). The Open Database License (ODbL) is a license agreement intended to allow users to freely share, modify, and use this Dataset while maintaining this same freedom for others, provided that the original source and author(s) are credited.Wan-Hao Chen, Hui-Lin Shu, Dan Li, Jian-Dong Liang, Xiu-Xiu Ren, Nalin N. Wijayawardene, Yan-Feng Han, Jie-Hong Zhao

Supplementary material 3 Aligement of LSU for dataset 1 Data type fas https://binary.pensoft.net/file/1441950This dataset is made available under the Open Database License (http://opendatacommons.org/licenses/odbl/1.0/). The Open Database License (ODbL) is a license agreement intended to allow users to freely share, modify, and use this Dataset while maintaining this same freedom for others, provided that the original source and author(s) are credited.Wan-Hao Chen, Hui-Lin Shu, Dan Li, Jian-Dong Liang, Xiu-Xiu Ren, Nalin N. Wijayawardene, Yan-Feng Han, Jie-Hong Zhao

Supplementary material 4 Aligement of RPB2 for dataset 1 Data type fas https://binary.pensoft.net/file/1441951This dataset is made available under the Open Database License (http://opendatacommons.org/licenses/odbl/1.0/). The Open Database License (ODbL) is a license agreement intended to allow users to freely share, modify, and use this Dataset while maintaining this same freedom for others, provided that the original source and author(s) are credited.Wan-Hao Chen, Hui-Lin Shu, Dan Li, Jian-Dong Liang, Xiu-Xiu Ren, Nalin N. Wijayawardene, Yan-Feng Han, Jie-Hong Zhao

Supplementary material 5 Aligement of tef-1a for dataset 1 Data type fas https://binary.pensoft.net/file/1441952This dataset is made available under the Open Database License (http://opendatacommons.org/licenses/odbl/1.0/). The Open Database License (ODbL) is a license agreement intended to allow users to freely share, modify, and use this Dataset while maintaining this same freedom for others, provided that the original source and author(s) are credited.Wan-Hao Chen, Hui-Lin Shu, Dan Li, Jian-Dong Liang, Xiu-Xiu Ren, Nalin N. Wijayawardene, Yan-Feng Han, Jie-Hong Zhao

Supplementary material 6 Dataset 1 for analysis 1 Data type nxs https://binary.pensoft.net/file/1441953This dataset is made available under the Open Database License (http://opendatacommons.org/licenses/odbl/1.0/). The Open Database License (ODbL) is a license agreement intended to allow users to freely share, modify, and use this Dataset while maintaining this same freedom for others, provided that the original source and author(s) are credited.Wan-Hao Chen, Hui-Lin Shu, Dan Li, Jian-Dong Liang, Xiu-Xiu Ren, Nalin N. Wijayawardene, Yan-Feng Han, Jie-Hong Zhao

Supplementary material 7 Aligement of ITS for dataset 2 Data type fas https://binary.pensoft.net/file/1441954This dataset is made available under the Open Database License (http://opendatacommons.org/licenses/odbl/1.0/). The Open Database License (ODbL) is a license agreement intended to allow users to freely share, modify, and use this Dataset while maintaining this same freedom for others, provided that the original source and author(s) are credited.Wan-Hao Chen, Hui-Lin Shu, Dan Li, Jian-Dong Liang, Xiu-Xiu Ren, Nalin N. Wijayawardene, Yan-Feng Han, Jie-Hong Zhao

Supplementary material 8 Aligement of LSU for dataset 2 Data type fas https://binary.pensoft.net/file/1441955This dataset is made available under the Open Database License (http://opendatacommons.org/licenses/odbl/1.0/). The Open Database License (ODbL) is a license agreement intended to allow users to freely share, modify, and use this Dataset while maintaining this same freedom for others, provided that the original source and author(s) are credited.Wan-Hao Chen, Hui-Lin Shu, Dan Li, Jian-Dong Liang, Xiu-Xiu Ren, Nalin N. Wijayawardene, Yan-Feng Han, Jie-Hong Zhao

Supplementary material 9 Aligement of RPB2 for dataset 2 Data type fas https://binary.pensoft.net/file/1441956This dataset is made available under the Open Database License (http://opendatacommons.org/licenses/odbl/1.0/). The Open Database License (ODbL) is a license agreement intended to allow users to freely share, modify, and use this Dataset while maintaining this same freedom for others, provided that the original source and author(s) are credited.Wan-Hao Chen, Hui-Lin Shu, Dan Li, Jian-Dong Liang, Xiu-Xiu Ren, Nalin N. Wijayawardene, Yan-Feng Han, Jie-Hong Zhao

Supplementary material 10 Aligement of tef-1a for dataset 2 Data type fas https://binary.pensoft.net/file/1441957This dataset is made available under the Open Database License (http://opendatacommons.org/licenses/odbl/1.0/). The Open Database License (ODbL) is a license agreement intended to allow users to freely share, modify, and use this Dataset while maintaining this same freedom for others, provided that the original source and author(s) are credited.Wan-Hao Chen, Hui-Lin Shu, Dan Li, Jian-Dong Liang, Xiu-Xiu Ren, Nalin N. Wijayawardene, Yan-Feng Han, Jie-Hong Zhao

Supplementary material 11 Dataset 2 for analysis 2 Data type nxs https://binary.pensoft.net/file/1441958This dataset is made available under the Open Database License (http://opendatacommons.org/licenses/odbl/1.0/). The Open Database License (ODbL) is a license agreement intended to allow users to freely share, modify, and use this Dataset while maintaining this same freedom for others, provided that the original source and author(s) are credited.Wan-Hao Chen, Hui-Lin Shu, Dan Li, Jian-Dong Liang, Xiu-Xiu Ren, Nalin N. Wijayawardene, Yan-Feng Han, Jie-Hong Zhao

Supplementary material 12 Aligement of ITS for dataset 3 Data type fas https://binary.pensoft.net/file/1441959This dataset is made available under the Open Database License (http://opendatacommons.org/licenses/odbl/1.0/). The Open Database License (ODbL) is a license agreement intended to allow users to freely share, modify, and use this Dataset while maintaining this same freedom for others, provided that the original source and author(s) are credited.Wan-Hao Chen, Hui-Lin Shu, Dan Li, Jian-Dong Liang, Xiu-Xiu Ren, Nalin N. Wijayawardene, Yan-Feng Han, Jie-Hong Zhao

Supplementary material 13 Aligement of LSU for dataset 3 Data type fas https://binary.pensoft.net/file/1441960This dataset is made available under the Open Database License (http://opendatacommons.org/licenses/odbl/1.0/). The Open Database License (ODbL) is a license agreement intended to allow users to freely share, modify, and use this Dataset while maintaining this same freedom for others, provided that the original source and author(s) are credited.Wan-Hao Chen, Hui-Lin Shu, Dan Li, Jian-Dong Liang, Xiu-Xiu Ren, Nalin N. Wijayawardene, Yan-Feng Han, Jie-Hong Zhao

Supplementary material 14 Aligement of RPB2 for dataset 3 Data type fas https://binary.pensoft.net/file/1441961This dataset is made available under the Open Database License (http://opendatacommons.org/licenses/odbl/1.0/). The Open Database License (ODbL) is a license agreement intended to allow users to freely share, modify, and use this Dataset while maintaining this same freedom for others, provided that the original source and author(s) are credited.Wan-Hao Chen, Hui-Lin Shu, Dan Li, Jian-Dong Liang, Xiu-Xiu Ren, Nalin N. Wijayawardene, Yan-Feng Han, Jie-Hong Zhao

Supplementary material 15 Aligement of tef-1a for dataset 3 Data type fas https://binary.pensoft.net/file/1441962This dataset is made available under the Open Database License (http://opendatacommons.org/licenses/odbl/1.0/). The Open Database License (ODbL) is a license agreement intended to allow users to freely share, modify, and use this Dataset while maintaining this same freedom for others, provided that the original source and author(s) are credited.Wan-Hao Chen, Hui-Lin Shu, Dan Li, Jian-Dong Liang, Xiu-Xiu Ren, Nalin N. Wijayawardene, Yan-Feng Han, Jie-Hong Zhao

Supplementary material 16 Dataset 3 for analysis 3 Data type nxs https://binary.pensoft.net/file/1441963This dataset is made available under the Open Database License (http://opendatacommons.org/licenses/odbl/1.0/). The Open Database License (ODbL) is a license agreement intended to allow users to freely share, modify, and use this Dataset while maintaining this same freedom for others, provided that the original source and author(s) are credited.Wan-Hao Chen, Hui-Lin Shu, Dan Li, Jian-Dong Liang, Xiu-Xiu Ren, Nalin N. Wijayawardene, Yan-Feng Han, Jie-Hong Zhao

Supplementary material 17 Aligement of ITS for dataset 4 Data type fas https://binary.pensoft.net/file/1441964This dataset is made available under the Open Database License (http://opendatacommons.org/licenses/odbl/1.0/). The Open Database License (ODbL) is a license agreement intended to allow users to freely share, modify, and use this Dataset while maintaining this same freedom for others, provided that the original source and author(s) are credited.Wan-Hao Chen, Hui-Lin Shu, Dan Li, Jian-Dong Liang, Xiu-Xiu Ren, Nalin N. Wijayawardene, Yan-Feng Han, Jie-Hong Zhao

Supplementary material 18 Aligement of LSU for dataset 4 Data type fas https://binary.pensoft.net/file/1441965This dataset is made available under the Open Database License (http://opendatacommons.org/licenses/odbl/1.0/). The Open Database License (ODbL) is a license agreement intended to allow users to freely share, modify, and use this Dataset while maintaining this same freedom for others, provided that the original source and author(s) are credited.Wan-Hao Chen, Hui-Lin Shu, Dan Li, Jian-Dong Liang, Xiu-Xiu Ren, Nalin N. Wijayawardene, Yan-Feng Han, Jie-Hong Zhao

Supplementary material 19 Aligement of RPB2 for dataset 4 Data type fas https://binary.pensoft.net/file/1441966This dataset is made available under the Open Database License (http://opendatacommons.org/licenses/odbl/1.0/). The Open Database License (ODbL) is a license agreement intended to allow users to freely share, modify, and use this Dataset while maintaining this same freedom for others, provided that the original source and author(s) are credited.Wan-Hao Chen, Hui-Lin Shu, Dan Li, Jian-Dong Liang, Xiu-Xiu Ren, Nalin N. Wijayawardene, Yan-Feng Han, Jie-Hong Zhao

Supplementary material 20 Aligement of tef-1a for dataset 4 Data type fas https://binary.pensoft.net/file/1441967This dataset is made available under the Open Database License (http://opendatacommons.org/licenses/odbl/1.0/). The Open Database License (ODbL) is a license agreement intended to allow users to freely share, modify, and use this Dataset while maintaining this same freedom for others, provided that the original source and author(s) are credited.Wan-Hao Chen, Hui-Lin Shu, Dan Li, Jian-Dong Liang, Xiu-Xiu Ren, Nalin N. Wijayawardene, Yan-Feng Han, Jie-Hong Zhao

Supplementary material 21 Dataset 4 for analysis 4 Data type nxs https://binary.pensoft.net/file/1441968This dataset is made available under the Open Database License (http://opendatacommons.org/licenses/odbl/1.0/). The Open Database License (ODbL) is a license agreement intended to allow users to freely share, modify, and use this Dataset while maintaining this same freedom for others, provided that the original source and author(s) are credited.Wan-Hao Chen, Hui-Lin Shu, Dan Li, Jian-Dong Liang, Xiu-Xiu Ren, Nalin N. Wijayawardene, Yan-Feng Han, Jie-Hong Zhao
