# Supplementary material for: ﻿Morphological and phylogenetic evidence reveals three new arthropod-associated species of Hypocreales (Clavicipitaceae, Bionectriaceae, and Myrotheciomycetaceae) from karst habitats in Guizhou, China
Source: MycoKeys. 2025 Oct 17;123:319–53. doi: 10.3897/mycokeys.123.164334 (PMC12552844; doi:10.3897/mycokeys.123.164334)
Supplement: Supplementary material 1 — List of recent studies reported new arthropods-associated fungi in nine families [file mycokeys-123-319-s001.docx]

Table S1 List of recent studies reported new arthropods-associated fungi in nine families

| Families | Species | Host/substrate | References |
| --- | --- | --- | --- |
| Bionectriaceae | *Ovicillium sinense* | Pupa | Chen et al. 2024a |
|  | *Biconidium sinense* | Soil | Wang et al. 2025 |
| Calcarisporiaceae | *Calcarisporium guizhouense* | fruiting body of *Cordyceps* sp. | Chen et al. 2024b |
| Clavicipitaceae | *Neoaraneomyces araneicola* | Spider | Chen et al. 2022a |
|  | *Pseudometarhizium araneogenum* | Spider | Chen et al. 2022a |
|  | *Pseudometarhizium lepidopterorum* | Pupa | Chen et al. 2022a |
| Cordycipitaceae | *Akanthomyces baishanensis* | Moth | Bu et al. 2025 |
|  | *Pleurodesmospora sanduensis* | Adult of Lepidoptera | Bu et al. 2025 |
|  | *Samsoniella duyunensis* | Ant | Chen et al. 2023 |
|  | *Samsoniella lurida* | Cocoon | Bu et al. 2025 |
|  | *Samsoniella torquatistipitata* | Ant | Bu et al. 2025 |
|  | *Samsoniella vallis* | Pupa | Chen et al. 2023 |
| Hypocreaceae | *Trichoderma strophariensis* | Mushroom | Tarafder et al. 2024 |
|  | *Trichoderma viridistromatis* | Mushroom | Tarafder et al. 2024 |
| Nectriaceae | *Bisifusarium keratinophilum* | Soil | Wang et al. 2024 |
| Ophiocordycipitaceae | *Purpureocillium zongqii* | Soil | Chen et al. 2024c |
|  | *Ophiocordyceps fenggangensis* | Larva | Peng et al. 2024 |
|  | *Ophiocordyceps liangii* | Larva | Peng et al. 2024 |
|  | *Ophiocordyceps musicaudata* | Larva | Peng et al. 2024 |
|  | *Ophiocordyceps sinocampes* | Larva | Xu et al. 2025 |
|  | *Ophiocordyceps cystidiata* | Larva | Xu et al. 2025 |
| Polycephalomycetaceae | *Perennicordyceps zongqii* | Moth | Chen et al. 2024c |
| Tilachlidiaceae | *Tilachlidium poneraticum* | Ant | Liang et al. 2016 |
